# Analysis of the Kuznets curve relationship between economic development and ecological environment in Aba Prefecture

**DOI:** 10.1371/journal.pone.0319929

**Published:** 2025-03-17

**Authors:** Xinglin Liu, Jing Zhou

**Affiliations:** School of Management, Chengdu University of Traditional Chinese Medicine, Chengdu, China; University of Kalyani, INDIA

## Abstract

**Background:**

The Yangtze and Yellow River basins play a critical role in China’s economic and social development as well as ecological security. As a key ecological barrier in the upper reaches of these rivers, Aba Prefecture has faced questions about whether its economic development after the 2008 earthquake has impacted its ecological environment.

**Objective:**

This study uses the Environmental Kuznets Curve (EKC) to analyze the relationship and characteristics between per capita GDP and the ecological environment in Aba Prefecture from 2010 to 2021. The aim is to provide scientific evidence for promoting the coordinated development of the economy and environment.

**Methods:**

The analysis employed the ADF test, Engle-Granger cointegration analysis, and correlation tests on the data. Finally, regression curve fitting was used to derive the relationship between per capita GDP and key environmental indicators in Aba Prefecture.

**Results:**

Per capita GDP in Aba Prefecture is highly negatively correlated with industrial wastewater discharge, highly positively correlated with domestic sewage discharge, positively correlated with industrial solid waste generation, and uncorrelated with industrial dust emissions. There exists a long-term equilibrium relationship between per capita GDP and both industrial wastewater discharge and industrial solid waste generation, indicating mutual influence and co-variation over time. The EKC curve in Aba Prefecture does not fully align with the traditional EKC model. Specifically, the relationship between per capita GDP and industrial wastewater discharge exhibits an “N” shape, while the relationships with industrial solid waste generation and domestic sewage discharge are linear and upward. There is no relationship between per capita GDP and industrial dust emissions.

**Conclusion:**

The relationship between the economy and the environment in Aba Prefecture exhibits a certain level of complexity. To achieve a long-term win-win outcome of both economic growth and environmental protection, it is essential to further deepen the transformation of the industrial structure, strengthen environmental governance measures, and optimize policy implementation.

## 1. Introduction

China has achieved rapid economic growth through clear economic development targets. By 2020, China’s GDP exceeded 100 trillion yuan, accounting for 17.3% of global GDP [1]. This rapid growth relies heavily on substantial investments, often leading to excessive resource consumption. Consequently, the ecological environment system faces unprecedented crises, exacerbating conflicts between economic development and environmental quality. Quantifying the relationship between economic growth and environmental quality and uncovering the underlying mechanisms have thus become focal points in academic research. The introduction of theories and methods such as Granger Causality [2], the Environmental Kuznets Curve (EKC) [3], Decoupling [4], Ecoefficiency [5], and Coupling Coordination [6] has provided robust theoretical guidance and quantitative tools for regional sustainable development. Given the complexity, mobility, and diffusivity of environmental resources, the EKC theory offers valuable empirical insights at a macro level.

The Kuznets Curve hypothesis, first proposed in 1955, reveals an inverted “U”-shaped relationship between per capita income and income inequality [7]. As economic productivity increases, Grossman and Krueger (1991) further posited that environmental degradation follows a similar inverted “U”-shaped relationship with per capita income, giving rise to the Environmental Kuznets Curve (EKC). This curve suggests that in the early stages of a country’s or region’s economic development, environmental pollution is relatively mild. However, as economic growth accelerates, environmental pollution intensifies. Once economic development reaches a certain threshold or turning point, pollution begins to decline with further increases in per capita income, leading to gradual improvements in environmental quality. This theory also demonstrates the existence of an inverted “U”-shaped relationship between air pollution and economic growth [8].

Pan Yuling (2023) highlighted that China’s ecological Kuznets Curve exhibits a “U”-shaped relationship overall. Specifically, the ecological Kuznets Curve for urban agglomerations follows an “N” shape, the curve for core cities is “U”-shaped, and the ecological conditions in other cities show a monotonous decline as economic levels rise [9]. Chen Yizhong (2024) [10] analyzed the correlation between green development and the environmental footprint in three typical mega-urban agglomerations along the Yangtze River. The study revealed that the correlation between green development levels and environmental footprints primarily follows an inverted “U” or inverted “N” EKC pattern. Liu Luofu (2024) [11] used Northam’s three-stage urbanization theory and the EKC hypothesis to explain the relationship between population changes and the urban heat island effect in Northeast China and the Yangtze River Delta region. Cheng Yan (2024) [12] applied K-means clustering to analyze the relationship between resource environments and socio-economic development, pointing out that most areas in the Yellow River Basin suffer from imbalances in resource-environment management and socio-economic development.

In summary, existing studies in China regarding the relationship between the economy and the environment in the Yangtze and Yellow River regions have mainly focused on large urban agglomerations, with limited research on the economic-environmental relationship in upstream counties. Aba Prefecture, as a critical water source conservation area for both the Yangtze and Yellow Rivers, plays an irreplaceable role in water and soil conservation, biodiversity protection, climate regulation, ecological security, and the sustainable development of the economy and society. Particularly, during the 2008 earthquake, Aba’s 13 counties, 215 townships, and 693,000 people were affected, with over 20,000 casualties, 45,100 injuries, severe infrastructure damage, a devastated industry, and significant ecological destruction, leading to direct economic losses of 90.27 billion yuan. After the earthquake, whether the ecology has recovered, whether the economy has developed, and what the relationship between ecology and economy is remain key questions worthy of exploration. This study, based on the actual situation of Aba Prefecture in Sichuan Province, selects relevant evaluation indicators of ecological environment and economic development from 2010 to 2021. The data are tested using ADF test, Engle-Granger cointegration analysis, and correlation analysis, followed by fitting curves to analyze the relationship between per capita GDP and key environmental indicators in Aba Prefecture. The aim is to assess the relationship between economic development and ecological environment.

The remainder of the paper is organized as follows: Section 2 provides an overview of the economic and environmental development in Aba Prefecture, detailing its geographical location and offering a summary of its economic and environmental status. Section 3 introduces the theoretical mechanism underlying the research, providing a conceptual framework for understanding the relationships examined. Section 4 outlines the research methods, including model selection, indicator selection, data sources, and the overall research framework. Section 5 presents the data analysis, starting with an overview of the data set, followed by model testing, including the unit root and cointegration tests, and a detailed analysis of the curve-fitting results. Section 6 provides the conclusion and discussion, summarizing the main findings and interpreting their implications. Finally, Section 7 discusses recommendations and limitations, offering policy suggestions and identifying avenues for future research.

## 2. Economic and environmental development in Aba Prefecture

### 2.1 Geographical location of Aba Prefecture

Aba Prefecture is located in the northwest of Sichuan Province, at the intersection of Sichuan, Gansu, and Qinghai provinces [13]. Situated on the southeastern edge of the Qinghai-Tibet Plateau, it has an average elevation of over 3,000 meters. The total area of Aba Prefecture spans 8.3003 million hectares, making it a critical ecological barrier and water source conservation area in the upper reaches of the Yangtze and Yellow Rivers.As of 2021, Aba Prefecture had a registered population of 897,200, comprising 535,400 Tibetans, 166,900 Qiang people, 27,900 Hui people, 165,300 Han people, and 1,700 individuals from other ethnic groups.

### 2.2 Economic and environmental overview of Aba Prefecture

By 2021, Aba Prefecture’s gross regional product (GDP) reached 44.963 billion yuan. Specifically, the added value of the primary, secondary, and tertiary industries was 8.83 billion yuan, 10.813 billion yuan, and 25.32 billion yuan, respectively, representing year-on-year growth rates of 6.9%, 8.3%, and 7.3% compared to 2020. The industrial structure was distributed as 19.6:24.0:56.4, and the per capita GDP was 54,900 yuan. During the “13th Five-Year Plan” period (2016-2020), the annual growth rate of industrial added value above the designated scale in Aba Prefecture averaged 4.2% (including two enclave industrial parks). During this time, the prefecture’s enterprises above the designated scale achieved an average annual operating income of 18.495 billion yuan, an increase of 4.162 billion yuan compared to the “12th Five-Year Plan” period (2011-2015). The average annual profit reached 1.034 billion yuan, an increase of 246 million yuan compared to the “12th Five-Year Plan.” The profit growth rate shifted from a 12.7% decline during the “12th Five-Year Plan” to a 17% increase during the “13th Five-Year Plan.” Per capita GDP rose significantly from 14,116 yuan in 2010 to 54,900 yuan in 2021.

The discharge of industrial wastewater in Aba Prefecture decreased significantly from 7.7838 million tons in 2010 to 1.2178 million tons in 2021. However, industrial soot emissions rose from 0.0143 million tons in 2010 to 0.5397 million tons in 2021. Similarly, the production of industrial solid waste increased from 221,070 tons in 2010 to 592,632.15 tons in 2021, and the discharge of domestic sewage grew from 12.2421 million tons in 2010 to 33.21119 million tons in 2021.

These data indicate that while Aba Prefecture has achieved rapid economic growth, the emissions of industrial soot, industrial solid waste, and domestic sewage continue to increase. This underscores the need for further improvements in the region’s environmental management capabilities.

## 3. Theoretical mechanism

The relationship between economic growth and environmental pressures is a central topic in regional sustainable development research. Economic growth reflects the wealth created by human utilization of environmental resources and serves as a key indicator of human development and well-being. One of the core theories in studying the economic-environment relationship is the Environmental Kuznets Curve (EKC) hypothesis. The EKC suggests an inverted “U”-shaped relationship between economic growth and environmental pollution. In the early stages of economic development, pollution often intensifies due to excessive reliance on natural resources and inefficient production technologies. However, as economic development reaches a certain threshold, structural transformations in industries, technological advancements, and growing public demand for better environmental quality lead to gradual improvements in environmental conditions. Theories of economic growth and sustainable development emphasize that technological progress [14] and capital accumulation [15] are the core drivers of economic growth and significantly influence the environmental pressures of economic activities. Moreover, environmental pollution, as a negative externality, requires correction through market mechanisms or policy interventions [16]. Examples include the promotion of technological innovation [17], the use of clean energy [18], and the development of green technologies [19]. Policy measures, such as environmental taxes [20], emissions trading, and environmental regulations [21], directly reduce pollution. Additionally, changes in public environmental awareness and consumption behaviors contribute to environmental improvements [22]. Green economy theories further emphasize that the development of clean energy, circular economies, and green finance can achieve a win-win situation for economic growth and environmental protection. This serves as an extension of the EKC theory.

**Fig 1:**
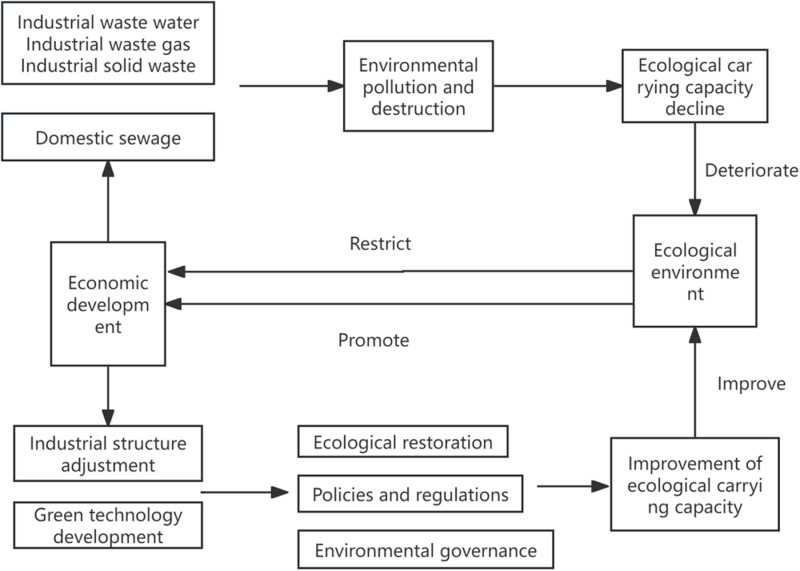
Theoretical mechanism of the relationship between economy and environment.

The complex interaction between economic development and the ecological environment can be analyzed through the lens of the EKC and related economic theories. Economic development has a dual impact on the environment. During the initial stages of economic growth and ecological degradation, the discharge of industrial wastewater, exhaust gases, solid waste, and domestic sewage increases, directly leading to pollution and ecological damage. This process reflects the upward phase of the EKC, where economic growth heavily relies on resource exploitation and high-pollution industrial production, resulting in a gradual decline in the carrying capacity of the ecological environment. As economic development progresses, industrial restructuring and the promotion of green technologies strengthen gradually. Economic growth not only drives technological innovation but also enhances societal environmental awareness and policy implementation efforts, ultimately leading to the restoration and improvement of the ecological environment. This corresponds to the turning point and downward phase of the EKC. The ecological environment also reacts to economic development. When pollution and ecological degradation reach a critical level, the decline in ecological carrying capacity begins to limit economic activities. Examples include resource depletion and health issues caused by environmental degradation, which can reduce productivity. The “constraint” arrows in Fig 1 visually illustrate the restrictive effects of ecosystems on economic development. Through ecological restoration, policy regulations, and environmental management measures, the carrying capacity of ecosystems can gradually improve. Enhanced ecological environments, in turn, provide a robust resource base and healthy conditions for economic activities, forming a positive feedback loop for economic growth. Policy regulations, environmental management, and ecological restoration play central roles in improving ecological carrying capacity and environmental quality. The innovation and promotion of green technologies significantly enhance resource utilization efficiency and pollution control. The framework illustrated in Fig 1 integrates the multidimensional relationships among economic growth, ecological environment changes, policy interventions, and technological advancements, systematically reflecting the dynamic interaction between economic growth and environmental quality.

## 4. Research methods

### 4.1 Model selection

The Environmental Kuznets Curve (EKC) has been validated in various studies, and simplified models are commonly employed in both domestic and international research. The simplified EKC equations are as follows (Soumyananda Dinda, 2004) [23]:


$$Y_{it}=\alpha_{i}+\beta_{1}x_{it}+\beta_{2}x_{it}{}^{2}+\beta_{3}x_{it}{}^{3}+\beta_{4}z_{it}+\varepsilon$$ (1)

$$Y_{it}=\alpha_{i}+\beta_{1}Inx$$ (2)

Where: y: Environmental degradation variable, x: Income, i: Study region, t: Time, β1, β2, β3, β4: Parameters to be estimated, ε: Random error term, z: Other factors influencing the environment, such as technology, geographic location, population density, and economic structure. The relationship between the environment and economic development can be categorized into the following six types:

1. β1 >  0, β2 =  β3 =  0: y increases linearly with x.
2. β1 <  0, β2 =  β3 =  0: y decreases linearly with x.
3. β1 >  0, β2 <  0, β3 =  0: y and x exhibit an inverted ‘U’-shaped relationship.
4. β1 <  0, β2 <  0, β3 =  0: y and x exhibit a ‘U’-shaped relationship.
5. β1 >  0, β2 <  0, β3 >  0: y and x exhibit an ‘N’-shaped relationship.
6. β1 <  0, β2 >  0, β3 <  0: y and x exhibit an inverted ‘N’-shaped relationship.

In Equation 1, the higher-order terms of income (x_it_^2^ and x_it_^3^) are designed to test potential nonlinear relationships between income and environmental degradation, such as “U-shaped” or “N-shaped” patterns, which are consistent with the theoretical framework of the Environmental Kuznets Curve (EKC) hypothesis. In contrast, Equation 2 provides a simplified representation based on a logarithmic transformation to describe the relationship between income and environmental degradation. This logarithmic form may better accommodate certain characteristics of empirical data, offering an alternative perspective for analysis. By using Equation 2 as a baseline linear model, a comparison with the nonlinear model in Equation 1 allows for the assessment of whether including higher-order terms significantly improves the explanatory power of the model. Such a comparison is critical for understanding the complexity of the relationship between economic development and environmental impacts.

### 4.2 Indicator selection

The EKC indicators consist of economic and environmental indicators. Economic development is typically measured using per capita GDP, a common metric for assessing the economic level of a country or region [24]. Environmental indicators generally include atmospheric indicators [25], water pollution indicators [26], solid waste indicators [27], ecological damage indicators [28], and other factors such as sanitation deficiencies [29]. Zhou Yangpin used the EKC curve to study Guangzhou’s urban environmental development trends by analyzing indicators such as industrial wastewater, industrial exhaust gases, industrial solid waste, domestic sewage, total suspended particles (TSP), and dust fall relative to per capita GDP. The findings indicated that economic growth does not automatically lead to environmental improvement; improvements only occur during the “post-management” stage [30]. Liu Tingting selected industrial exhaust gas emissions, industrial wastewater discharge, and industrial solid waste generation from the same period to represent environmental pollution levels, along with domestic sewage discharge and per capita GDP, and concluded that Ningxia’s industrial sector from 1991 to 2007 was still in an upward phase, with no EKC turning point for pollutant emissions yet reached [31]. Xu Fangjin examined the relationship between urbanization and pollutant emissions (including industrial wastewater, industrial SO₂, industrial soot, and domestic waste) to determine whether an EKC relationship exists [32]. Considering the unique post-earthquake conditions in Aba Prefecture, this study analyzes trends in ecological environment changes from perspectives such as ecological importance, ecosystem vulnerability, environmental capacity, and environmental economics. For environmental indicators, we selected industrial wastewater discharge, industrial soot emissions, industrial solid waste generation, and domestic sewage discharge. Per capita GDP was chosen as the indicator to measure economic growth.

### 4.3 Data sources

The data primarily came from the *China Statistical Yearbook* , *China Environmental Statistical Yearbook* , *Aba Prefecture Yearbook* , public reports from the Aba Prefecture Ecological Environment Bureau, and data obtained through applications to relevant departments of the Aba Prefecture government.

### 4.4 Research framework

A unit root test is conducted on the time-series data to determine whether the series is stationary. If the series is stationary, regression methods can be directly used for curve fitting. If the series is non-stationary (i.e., contains a unit root), differeneing is performed. When the series becomes stationary after the i-th differencing (up to two times), it is considered integrated of order i. If all tested sequences were integrated to the same order, a cointegration test was conducted to assess whether there was a long-term equilibrium relationship among variables. If such a relationship was present, curve fitting regression could then be applied to the time-series data.

## 5. Data analysis

### 5.1 Overview

As shown in Table 1, the data spans 12 years, from 2010 to 2021. The discharge of industrial wastewater peaked at 10.9906 million tons in 2011 and reached its lowest value of 1.2178 million tons in 2020, showing a significant downward trend. The average industrial soot emission was 0.5635 million tons, with a minimum of 0.0143 million tons and a maximum of 1.0034 million tons in 2015. The standard deviation of 0.2891 million tons indicates that soot emissions were relatively stable with minor fluctuations. The average production of industrial solid waste was 5.599 million tons, with a minimum of 2.2107 million tons in 2010 and a maximum of 12.85382 million tons in 2020, demonstrating a notable increase. Domestic sewage discharge had an average of 20.6966 million tons and showed an annual increasing trend. Meanwhile, per capita GDP displayed steady growth over the period.

**Table 1:** Basic situation of environment and economic development from 2010 to 2021.

Year	Industrial wastewater discharge (million tons)	Industrial fume emissions (million tons)	Industrial solid waste generation (million tons)	Domestic sewage discharge (million tons)	GDP per capita (yuan)
2010	778.380	0.014	22.107	1224.210	14116
2011	1099.065	0.251	47.928	1667.720	17738
2012	1037.826	0.234	45.594	1752.867	21180
2013	1020.701	0.232	45.778	1866.200	24373
2014	933.728	0.937	46.653	1954.993	26875
2015	945.248	1.003	49.360	2104.120	31284
2016	876.728	0.782	53.190	2368.570	33432
2017	609.204	0.669	82.281	2511.268	36864
2018	526.315	0.669	83.211	2632.619	43220
2019	405.422	0.572	100.627	2383.022	46376
2020	124.736	0.638	128.538	3078.235	49668
2021	121.780	0.540	59.263	3321.119	54900

Data Source: Aba Tibetan and Qiang Autonomous Prefecture Ecological Environment Bureau, * Aba Yearbook * (2010–2021).

### 5.2 Model testing

To avoid spurious regression, the stationarity of variables must be tested before performing time series regression [33]. The standard methods for testing time series stationarity include the Augmented Dickey-Fuller (ADF) test proposed by Dickey et al. [34], the Phillips-Perron (PP) test proposed by Phillips et al. [35], and the Kwiatkowski-Philips-Schmidt-Shin (KPSS) test [36]. Among these, the ADF test is considered more robust for small sample sizes [37]. Therefore, this study employed the ADF test to examine the stationarity of the data. Spurious regression is a common issue when directly analyzing non-stationary series. However, when specific linear combinations of non-stationary variables are stationary, an intrinsic equilibrium mechanism exists among these variables. While the individual variables may exhibit non-stationary behavior, the system as a whole is stationary, indicating a long-term equilibrium relationship. Cointegration testing is a prerequisite for identifying whether such a long-term equilibrium exists among the variables [38].

#### 5.2.1 Unit root test.

After taking the logarithm of the raw data, the ADF unit root test was performed. As shown in Table 2, the results indicate that the original data failed the unit root test, confirming that all series were non-stationary. Subsequently, a first-order differencing was applied, and the results revealed that only per capita GDP and industrial wastewater discharge (10,000 tons) passed the test. Following a second-order differencing, per capita GDP, industrial wastewater discharge, industrial soot emissions, industrial solid waste generation, and domestic sewage discharge achieved stationarity at the 5% and 1% significance levels (*P* <  0.05). This indicates that all variables are integrated of order two (*I*^*2*^), making them suitable for further cointegration analysis.

**Table 2:** Unit root test.

Variable	Difference Order	ADF Value	1%Critical Value	5%Critical Value	Conclusion
GDP per capita (yuan)	0	2.327	-4.421	-3.260	Non-stationary
GDP per capita (yuan)	1	-3.383	-4.297	-3.213	Stationary
GDP per capita (yuan)	2	-5.533	-4.583	-3.321	Stationary
Industrial wastewater discharge (million tons)	0	0.358	-4.200	-3.175	Non-stationary
Industrial wastewater discharge (million tons)	1	-4.582	-4.297	-3.213	Stationary
Industrial wastewater discharge (million tons)	2	-3.491	-4.583	-3.321	Stationary
Industrial fume emissions (million tons)	0	-2.146	-4.200	-3.175	Non-stationary
Industrial fume emissions (million tons)	1	-2.768	-4.297	-3.213	Non-stationary
Industrial fume emissions (million tons)	2	-3.529	-4.583	-3.321	Stationary
Industrial solid waste generation (million tons)	0	-1.973	-4.200	-3.175	Non-stationary
Industrial solid waste generation (million tons)	1	-2.967	-4.297	-3.213	Non-stationary
Industrial solid waste generation (million tons)	2	-3.258	-2.886	-1.996	Stationary
Domestic sewage discharge (million tons)	0	2.382	-2.792	-1.978	Non-stationary
Domestic sewage discharge (million tons)	1	-3.291	-4.583	-3.321	Non-stationary
Domestic sewage discharge (million tons)	2	-5.592	-4.421	-3.260	Stationary

#### 5.2.2 Cointegration test.

To avoid spurious regression[39], cointegration testing is required to examine whether there is a long-term equilibrium relationship among the variables before regression estimation[40]. This study employs the Engle-Granger cointegration test [41]to analyze the cointegration relationships between the second-order differenced variables, investigating whether a long-term stable relationship exists between economic development and environmental pollution in Aba Prefecture. According to the results in Table 3, the P-value of the ADF test for industrial wastewater discharge (10,000 tons) is 0.00095 ( < 0.05), rejecting the null hypothesis of a unit root. This indicates that the “industrial wastewater discharge” series is stationary. The stationarity and long-term stability characteristics suggest a cointegration relationship between industrial wastewater discharge and per capita GDP, implying a common trend over the long term. Similarly, the P-value of the ADF test for industrial solid waste generation is 0.02596 ( < 0.05), rejecting the null hypothesis of a unit root and confirming that the series is stationary. Additionally, this variable exhibits a cointegration relationship with per capita GDP, indicating a stable long-term relationship. Therefore, regression estimation can be performed for these variables.

**Table 3:** Engle-Granger cointegration relationship test.

Variable	ADF Value	P Value	Cointegration Relationship
Industrial wastewater discharge (million tons)	-4.104	0.001	Exists
Industrial fume emissions (million tons)	0.374	0.980	Does not exist
Industrial solid waste generation (million tons)	-3.108	0.026	Exists
Domestic sewage discharge (million tons)	0.598	0.988	Does not exist

This indicates that changes in industrial wastewater discharge and industrial solid waste generation are cointegrated with per capita GDP, suggesting that these variables may co-vary and influence each other over the long term. In contrast, the long-term equilibrium relationships between industrial soot emissions, domestic sewage discharge, and per capita GDP are weaker. Their variations may be more influenced by short-term factors or driven by different underlying mechanisms.

#### 5.2.3 Correlation test between per capita GDP and industrial “three wastes” as well as domestic sewage discharge.

The Pearson correlation method[42] was used to analyze the correlation between per capita GDP and industrial wastewater discharge, industrial soot emissions, industrial solid waste generation, and domestic sewage discharge in Aba Prefecture from 2010 to 2021. As shown in Table 4, under the significance level of (*P* <  0.01). Per capita GDP is highly negatively correlated with industrial wastewater discharge, indicating that as per capita GDP increases, industrial wastewater discharge decreases. Per capita GDP is highly positively correlated with domestic sewage discharge, meaning that an increase in per capita GDP corresponds to an increase in domestic sewage discharge. Per capita GDP is positively correlated with industrial solid waste generation, implying that as per capita GDP rises, industrial solid waste generation also increases. The correlation between per capita GDP and industrial soot emissions is not statistically significant (*P* =  0.11).

**Table 4:** Correlation test.

	GDP per capita (yuan)	Industrial wastewater discharge(million tons)	Industrial fume emissions (million tons)	Industrial solid waste generation (million tons)	Domestic sewage discharge (million tons)
GDP per capita (yuan) Pearson correlation	1	-.888**	0.485	.768**	.962**
Significance(Two-tailed)		0.000	0.11	0.004	0.000
Number of Variables	12	12	12	12	12

### 5.3 Analysis of curve fitting results

#### 5.3.1 EKC curve for industrial wastewater discharge.

**Fig 2:**
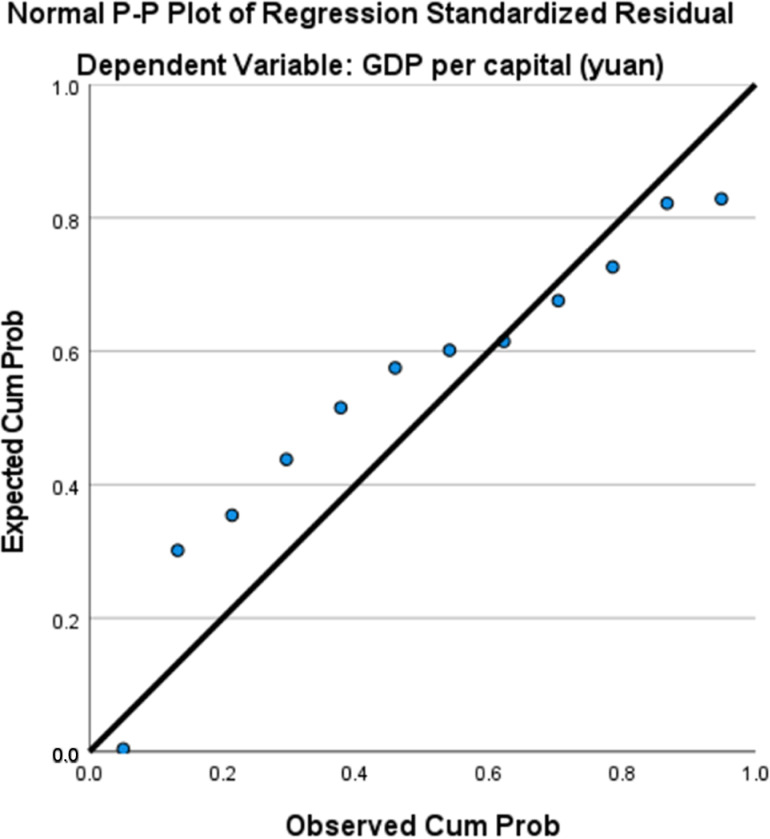
Residual map of industrial wastewater discharge in Aba Prefecture.

**Fig 3:**
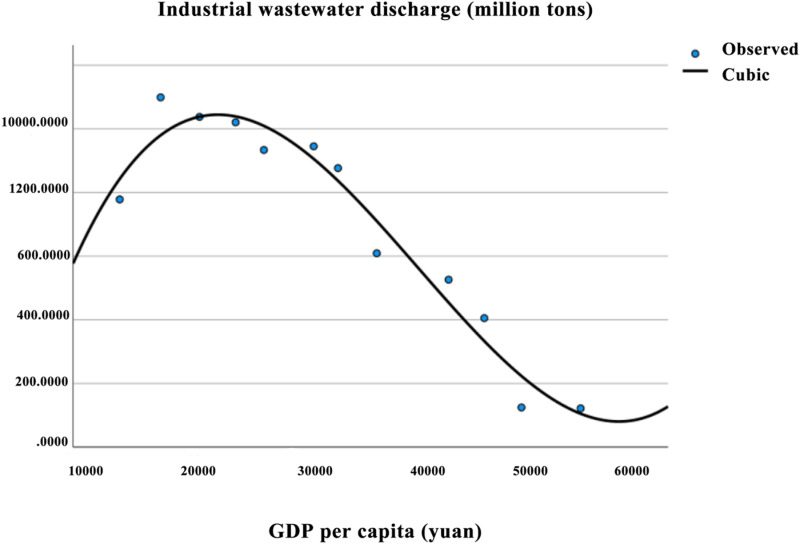
EKC curve of industrial wastewater discharge in Aba prefecture.

Based on the indicators of per capita GDP and industrial wastewater discharge, a curve regression simulation was performed using per capita GDP as the X-axis and industrial wastewater discharge as the Y-axis. The regression equations and curve fitting graphs were generated. Table 5 presents the regression coefficients and statistical significance of each regression equation. According to the statistical test results, all three models passed the F-test. However, the regression coefficient β_1_ in the quadratic model failed the T-test. The cubic model demonstrated the best fit, with an R^2^ value of 0.956, and was therefore selected as the final fitting model. Based on the residual plot, the distribution of the residuals in Fig 2 appears to be relatively random and uniform, with no significant deviation from the center, suggesting that the residuals roughly follow a normal distribution. By examining the pattern of residuals as they vary with the independent variables, and as shown in Table 6, where the Variance Inflation Factor (VIF) equals 1, it indicates that there is no linear correlation between the independent variables. This suggests that the regression model adequately meets the assumption of linearity and does not exhibit significant heteroscedasticity.The EKC regression equation for industrial wastewater discharge in Aba Prefecture is: y = -662.387 + 0.172X-5.251E-6X2 + 4.32E-11X3. The fitted curve is shown in Fig 3.

**Table 5:** Statistical test of fitting model of industrial wastewater discharge in Aba Prefecture.

Environmental Indicator	Curve Characteristic	Test Parameter			Model Coefficients			
		R^2^	F	sig	α	β_1_	β_2_	β_3_
Industrial wastewater discharge(million tons)	Logarithmic Function	0.639	17.688	0.002	7395.588	-647.178**	/	/
	Quadratic Function	0.913	46.943	0.000	652.035	0.033	-8.17E-07*	/
	Cubic Function	0.956	57.755	0.000	-662.387	0.172**	-5.25E-06**	4.32E-11**

Note:

**P <  0.01;

*  *P* <  0.05

**Table 6:** Coefficients of collinearity for industrial wastewater discharge.

Coefficients							
	Standardized Coefficients			95.0% Confidence Interval for B		Collinearity Statistics	
	Beta	t	Sig.	Lower Bound	Upper Bound	Tolerance	VIF
(Constant)		13.297	0	47582.533	66738.305		
Industrial wastewater discharge(million tons)	-0.888	-6.122	0	-45.99	-21.446	1	1

Dependent Variable: GDP per capital(yuan)

From the fitted curve of per capita GDP and industrial wastewater discharge in Fig 3, the cubic function reveals that β₁ >  0, β₂ <  0, and β₃ >  0, indicating an “N-shaped” curve for environmental pollution. The “N-shaped” relationship suggests that environmental degradation initially decreases to a certain level before rising again. This aligns with the “Reversal Hypothesis,” which posits that the separation between environmental degradation and economic growth will not persist in the long term, and after reaching a certain level, they will recombine. This is in contrast to the “inverted N-shaped” relationship between industrial wastewater discharge and per capita GDP found in Inner Mongolia, as observed by scholar Qiao Ting [43]. However, it is consistent with the N-shaped relationship observed in the industrial wastewater discharge in Sichuan Province, as studied by Guo Hui [44]. Given that Aba Prefecture is part of Sichuan Province and is an important ecological barrier on the Qinghai-Tibet Plateau, this research holds significant value. From Fig 3, a turning point appears in 2011, indicating that before 2011, industrial wastewater discharge increased with rising per capita GDP. However, after the economy reached a certain level, industrial wastewater discharge began to decrease. From 2011 to 2021, as GDP grew, industrial wastewater discharge showed a downward trend. After the 2008 earthquake, Aba Prefecture began its recovery, especially in 2010, a critical year of the 11th Five-Year Plan. Between 2010 and 2011, the rapid economic growth in Aba Prefecture led to a 40% increase in industrial wastewater discharge. In 2011, the Aba Prefecture government introduced the “2010 Pollution Source Census Dynamic Update Work Plan,” establishing a dedicated working group to adopt a “dynamic adjustment, key investigation, and overall accounting” approach. This included conducting targeted investigations for key enterprises and estimating pollution from non-key enterprises by proportion. The plan also set the total reduction base for Aba’s “12th Five-Year” period.In the same year, the “12th Five-Year Plan Outline for National Economic and Social Development of Aba Tibetan and Qiang Autonomous Prefecture” was released, with goals centered around “Ecological Aba, Charming Aba, Prosperous Aba, and Harmonious Aba.” The plan emphasized strengthening environmental infrastructure, pollution control in key industries and regions, and improving waste and sewage treatment capacity, reaching 150,000 cubic meters per year and 84,000 tons per day. The Aba section of the Minjiang and Dadu Rivers achieved the Class III water quality standard under the “Surface Water Environmental Quality Standards” (GB3838-2002), highlighting the growing ecological advantages.Subsequently, a series of regulations and policies were introduced, such as the “Aba Prefecture Western Sichuan Ecological Economic Demonstration Zone Construction Outline,” the “2016 Economic Work Key Points for the Prefecture,” the “2018 Annual Focus Work Arrangement on Legal Government Construction,” and the “Regulations on Ecological Environmental Protection of Aba Tibetan and Qiang Autonomous Prefecture.” These measures included strict implementation of environmental information disclosure, environmental impact assessments, and total pollutant discharge control, leading to a decline in the conflict between environmental protection and economic development in Aba Prefecture.

#### 5.3.2 EKC curve for industrial solid waste generation.

**Fig 4:**
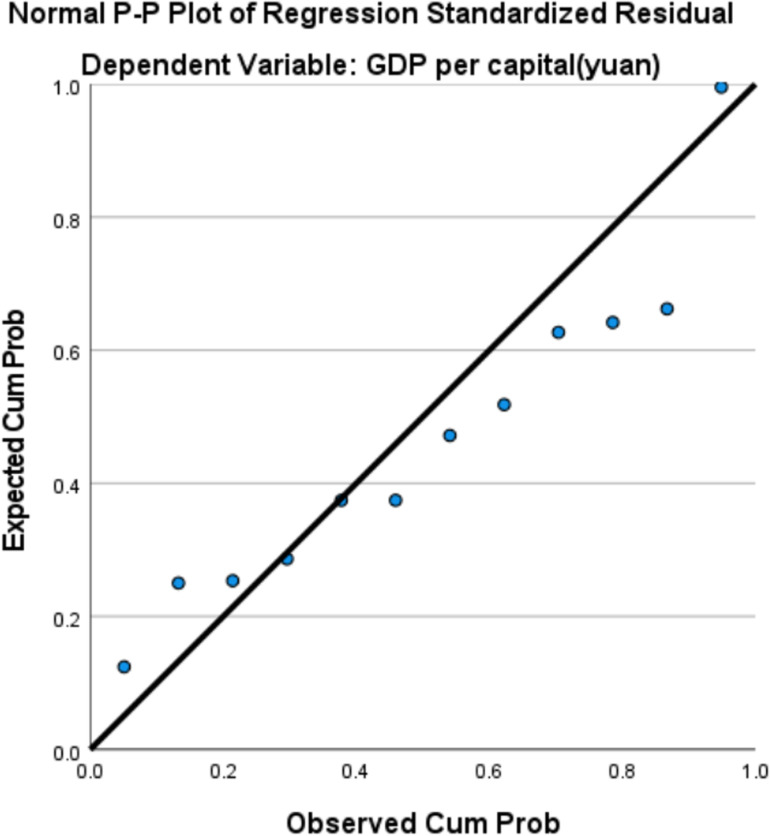
Residual map of industrial solid waste discharge in Aba Prefecture.

**Fig 5:**
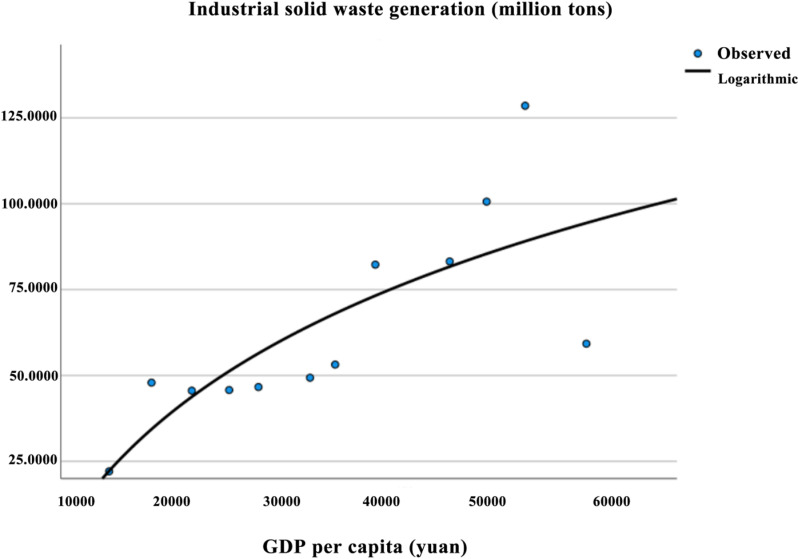
EKC curve of industrial solid waste emissions in Aba prefecture.

As shown in Table 7, all three models passed the F-test for the overall significance of the regression equations. However, in the quadratic and cubic models, the regression coefficients β1 and β2 failed the T-test. In contrast, all regression coefficients in the logarithmic model passed the T-test. Based on the residual plot (Fig 4), the residuals show good randomness and uniformity, with no significant deviation from the center, suggesting that the residuals roughly follow a normal distribution. By observing the pattern of residuals as they vary with the independent variables, and as shown in Table 8, where the Variance Inflation Factor (VIF) equals 1, it indicates that there is no linear correlation between the independent variables. This suggests that the regression model is likely free from serious issues of heteroscedasticity, non-linearity, or autocorrelation, and the residuals approximately conform to a normal distribution.Therefore, the logarithmic model was selected as the final fitting model. The EKC regression equation for industrial solid waste generation in Aba Prefecture is: y = -485.509 + 53.138InX. The fitted curve is shown in the corresponding Fig 5.

**Table 7:** Statistical test of fitting model of industrial solid waste in Aba Prefecture.

Environmental Indicator	Curve Characteristic	Test Parameter			Model Coefficients			
		R^2^	F	sig	α	β_1_	β_2_	β_3_
Industrial solid waste generation (million tons)	Logarithmic Function	0.595	14.671	0.003	-485.509	53.138**	/	/
	Quadratic Function	0.612	7.083	0.014	-23.973	0.004	-2.97E-08	/
	Cubic Function	0.729	7.168	0.012	160.094	-0.016	5.91E-07	-6.05E-12*

Note:

**P <  0.01;

*  *P* <  0.05

**Table 8:** Coefficients of collinearity for industrial solid waste generation.

Coefficients^a^							
	Standardized Coefficients			95.0% Confidence Interval for B		Collinearity Statistics	
	Beta	t	Sig.	Lower Bound	Upper Bound	Tolerance	VIF
(Constant)		1.83	0.097	-2504.614	25562.672		
Industrial solid waste generation (million tons)	0.768	3.786	0.004	140.859	543.687	1	1

^a^ Dependent Variable: GDP per capita (yuan)

From Fig 5, it can be observed that β₁ >  0, β₂ =  0, and β₃ =  0, indicating that the Environmental Kuznets Curve (EKC) for industrial solid waste emissions in Aba Prefecture follows a linear relationship and is increasing. The data show that from 2010 to 2020, industrial solid waste emissions exhibited an overall increasing trend, reaching a peak in 2020 without a turning point, indicating that Aba Prefecture remains in the “scale effect” stage. This finding is consistent with the research of Qiao Ting on Inner Mongolia [43], where no turning point was observed, but it contrasts with the findings of Zhai Xiaowei [45] and others [46], who observed a different trend in industrial solid waste emissions.Specifically, from 2010 to 2011, the emissions showed an increasing trend. In 2011, Aba Prefecture’s total industrial added value was 5.909 billion yuan, with a growth rate of 30.4%, contributing 61.4% to economic growth, and driving the economy up by 9.3 percentage points, with an industrialization rate of 35.1%. As a result, industrial solid waste emissions also increased sharply. From 2011 to 2012, the trend shifted to a decline due to the optimization and adjustment of Aba’s industrial structure. The GDP composition of the three industries was adjusted from the previous year’s 16.5:47.2:36.3 to 15.5:50.1:34.4, with decreases in the first and third industries by 1.0 and 1.9 percentage points, respectively. From 2012 to 2020, there was a general upward trend, with emissions reaching 1,285,382.2 tons in 2020. Notably, from 2019 to 2020, there was a sharp increase in emissions.The primary source of industrial solid waste emissions is the consumption of traditional energy in industrial production, indicating that Aba Prefecture’s industrial development is still dependent on the use of traditional energy sources and high-pollution, high-emission production methods. In 2019, the Aba Prefecture government held its 33rd executive meeting and proposed to intensify pollution control efforts and continue rectifying environmental issues. In 2020, a special meeting was held on the inspection and rectification of ecological and environmental protection issues, implementing a strict “list + responsibility + closure” system. As a result, emissions showed a slight decline from 2020 to 2021. Moreover, 2021 marked the beginning of the 14th Five-Year Plan, a key year for accelerating the development of the Aba Ecological Demonstration Zone in Northwestern Sichuan, which could signal a potential easing of the conflict between ecological protection and economic development in Aba Prefecture.

#### 5.3.3 EKC curve for domestic sewage discharge.

**Fig 6:**
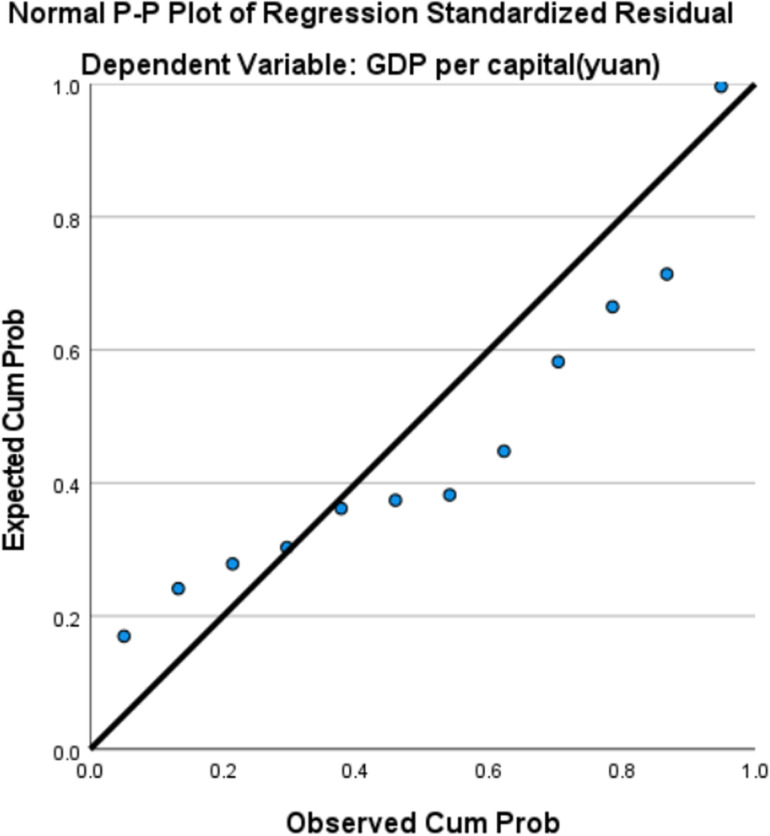
Residual map of domestic sewage discharge in Aba Prefecture.

**Fig 7:**
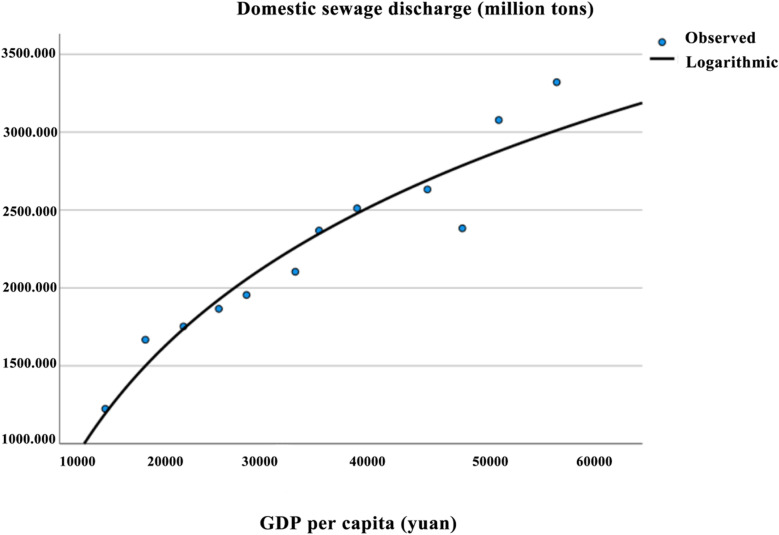
EKC curve of domestic sewage discharge in Aba prefecture.

As shown in Table 9, all three models passed the F-test for the overall significance of the regression equations. However, in the quadratic and cubic models, the regression coefficients β1 and β2 failed the T-test. In contrast, all regression coefficients in the logarithmic model passed the T-test. The residual plot in Fig 6 shows that the residual points are roughly uniformly distributed and exhibit no apparent deviation from the center, indicating that the residuals approximately follow a normal distribution. According to Table 10, the VIF value of 1 indicates an ideal situation, with no evidence of significant heteroscedasticity or non-linear relationships. This suggests that the regression model satisfies the assumptions of linearity and homoscedasticity, supporting the reliability of the model’s results.Therefore, the logarithmic model was selected as the final fitting model. The EKC regression equation for domestic sewage discharge in Aba Prefecture is: y = -11594.664 + 1338.419InX. The fitted curve is shown in the corresponding Fig 7.

**Table 10:** Coefficients of collinearity for domestic sewage discharge.

Coefficients^a^							
	Standardized Coefficients			95.0% Confidence Interval for B		Collinearity Statistics	
	Beta	t	Sig.	Lower Bound	Upper Bound	Tolerance	VIF
(Constant)		-3.161	0.01	-23459.277	-4059.384		
Domestic sewage discharge (million tons)	0.962	11.17	0	16.84	25.233	1	1

^a^ Dependent Variable: GDP per capital(yuan)

**Table 9:** Statistical test of domestic sewage discharge fitting model in Aba Prefecture.

Environmental Indicator	Curve Characteristic	Test Parameter			Model Coefficients			
		R^2^	F	sig	α	β_1_	β_2_	β_3_
Domestic sewage discharge (million tons)	Logarithmic Function	0.907	97.291	0.000	-11594.664	1338.419**		
	Quadratic Function	0.926	56.18	0.000	743.066	0.046	-2.80E-08	
	Cubic Function	0.946	46.331	0.000	-796.544	0.209	-5.22E-06	5.06E-11*

Note:

**P <  0.01;

*  *P* <  0.05

According to Table 10, it can be observed that β₁ >  0, β₂ =  0, and β₃ =  0, indicating that the Environmental Kuznets Curve (EKC) for domestic sewage discharge in Aba Prefecture follows a linear, upward relationship. From 2010 to 2021, the trend remained overall increasing without a turning point. This result is consistent with the research of Qiao Ting on Inner Mongolia [43], and with the findings of Leng Jianfei, who studied the relationship between per capita GDP and domestic sewage discharge as well as domestic NH₃-N emissions, which also showed no downward trend or turning point [47].Specifically, from 2010 to 2018, domestic sewage discharge exhibited an increasing trend. However, from 2018 to 2019, there was a slight decrease, followed by an upward trend from 2019 to 2021. The year 2019 marked the beginning of the comprehensive construction of the Aba Ecological Demonstration Zone in Northwestern Sichuan, as well as the first year of the Aba Ecological Environment Bureau’s new efforts and initiatives. With a focus on the “One Prefecture, Two Zones, Three Gardens” strategic goals, the conflict between ecological sewage discharge and economic development was somewhat alleviated during this period. However, after 2019, the increasing trend continued, highlighting the ongoing challenges in environmental governance regarding domestic sewage discharge in Aba Prefecture.

#### 5.3.4 EKC curve for industrial soot emissions.

According to Table 11, it can be observed that both the quadratic and cubic models in the EKC curve fitting are not statistically significant (P >  0.05), whereas the logarithmic model shows statistical significance. However, since the R² value is less than 0.5, it lacks explanatory power. This indicates that, during this period, the industrial dust emissions in Aba Prefecture were not significantly influenced by economic development. This finding is consistent with the results of Lv Yonglong’s study, which also suggests that industrial emissions are not closely tied to economic growth.In 2012, China proposed strengthening ecological civilization construction, and in 2015, the country implemented the strictest-ever Environmental Protection Law. With the implementation of these policies and regulations, it is foreseeable that the decoupling of economic growth and pollution emissions in China will become increasingly prominent. In Aba Prefecture, relevant regulations and policies, such as the Aba Prefecture 2010 Pollution Source Census Dynamic Update Work Plan, the Aba Tibetan and Qiang Autonomous Prefecture Wetland Protection Regulations, and the Aba Prefecture Northwestern Sichuan Ecological Economic Demonstration Zone Construction Outline were introduced in 2011. These measures focused on optimizing the energy structure, strictly controlling emissions from enterprises, promoting industrial upgrades, and developing emerging industries such as new energy and circular economy. These policies may have contributed to the reduction of industrial emissions, thereby weakening the direct relationship between emissions and economic growth.

**Table 11:** Statistical test of industrial soot emission fitting model in Aba Prefecture.

Environmental Indicator	Curve Characteristic	Test Parameter			Model Coefficients			
		R^2^	F	sig	α	β_1_	β_2_	β_3_
Industrial fume emissions (million tons)	Logarithmic Function	0.356	5.539	0.04	-3.857	0.426		
	Quadratic Function	0.657	8.617	0.008	-1.192	0	-1.33E-09	
	Cubic Function	0.676	5.554	0.023	-1.952	0	-3.90E-09	2.50E-14

#### 5.4.4 Robustness analysis of the regression model.

The primary purpose of robustness analysis is to ensure the stability of the regression model under different assumptions, data conditions, and methods[48]. By examining aspects such as heteroscedasticity, multicollinearity, outliers, model selection, and sample selection, the reliability and interpretability of the regression results can be enhanced. Common methods for robustness analysis include: Heteroscedasticity Tests: To detect whether the variance of errors is constant across observations. Multicollinearity Diagnosis: To identify and address high correlations among independent variables that may affect regression estimates. Outliers and Influential Points: To detect and manage extreme values or influential data points that could distort the regression results [49].

This regression model adopts **multicollinearity diagnosis**, a method commonly used to detect multicollinearity. Multicollinearity can be diagnosed using several approaches, including the Variance Inflation Factor (VIF)[50], eigenvalue decomposition, and Condition Index. In this study, the **Variance Inflation Factor (VIF)** method is used. VIF is one of the most widely used multicollinearity diagnostic indicators and reflects the extent to which the variance of each independent variable is inflated due to its correlation with other independent variables. Specifically, VIF measures the factor by which the variance of a variable increases because of multicollinearity with other variables. The calculation formula is as follows: VIF j = 1/1-R _j_^2^ Where R _j_^2^ is the squared correlation value of the independent variable X_j_ with all other independent variables R^2^ value in the regression model of X_j_.

VIF =  1: Indicates no linear correlation between the independent variable and other independent variables.
1 < VIF ≤  5: Indicates some correlation between the independent variable and other independent variables, but it does not cause severe problems.
VIF >  10: Indicates a high correlation between independent variables, suggesting a serious multicollinearity issue.

From Table 12, it can be observed that **domestic sewage discharge (10,000 tons)** has a moderate positive correlation with **per capita GDP**, with a VIF value of 5.586 ( > 5). However, since the VIF value is less than 10, and the VIF for domestic sewage discharge and per capita GDP is 1 (as shown in Table 10), it indicates that there is no multicollinearity issue for this variable, ensuring the stability of the regression coefficients. For the remaining variables, the VIF values are all less than 5, suggesting that the multicollinearity is minimal.

**Table 12:** Robustness analysis.

	Standardized Coefficient (Beta)	t	Significance	95% Confidence Interval		Collinearity statistics	
				Lower	Upper	Tolerance	VIF
constant		0.851	0.423	-17555.321	37294.76		
Discharge of domestic sewage (million tons)	0.567	3.237	0.014	3.342	21.462	0.179	5.586
Industrial wastewater discharge (million tons)	-0.341	-2.059	0.078	-27.789	1.92	0.201	4.983
Industrial soot emission (million tons)	0.119	1.209	0.266	-4908.456	15179.474	0.565	1.769
Production of industrial solid waste (million tons)	0.072	0.627	0.551	-88.815	152.869	0.419	2.388

## 6. Conclusion and discussion

### 6.1 Conclusion

This study systematically examined the relationship between per capita GDP and industrial “three wastes” (industrial wastewater, industrial soot, and industrial solid waste) as well as domestic sewage in Aba Prefecture from 2010 to 2021. Using time-series econometric tests, the existence and trends of EKC curves in the region were analyzed. The key findings are as follows:

Correlation Analysis: Per capita GDP in Aba Prefecture is highly negatively correlated with industrial wastewater discharge, highly positively correlated with domestic sewage discharge, positively correlated with industrial solid waste generation, and uncorrelated with industrial soot emissions.

Long-Term Equilibrium: There is a long-term equilibrium relationship between per capita GDP and both industrial wastewater discharge (10,000 tons) and industrial solid waste generation (10,000 tons). This suggests that these variables may co-vary and influence each other over time. For sustained long-term economic growth in Aba Prefecture, environmental pollution must be effectively controlled.

EKC Curve Specificity: The EKC curve for Aba Prefecture does not fully align with the traditional EKC model, demonstrating its unique characteristics. Specifically: Per capita GDP and industrial wastewater discharge exhibit an “N”-shaped relationship. Per capita GDP and both industrial solid waste generation and domestic sewage discharge show upward linear relationships. Industrial soot emissions are unrelated to overall economic development, suggesting they may be influenced primarily by specific industrial activities rather than the overall level of economic growth.

The downward trend in pollution levels beginning in 2020 indicates that adjustments to the industrial structure have alleviated some conflicts between economic growth and the ecological environment. However, environmental governance and pollution control in Aba Prefecture remain significant challenges.

### 6.2 Discussion

With the rapid economic development, industrial structure [51] and policy management [52] are crucial for driving economic growth and achieving sustainable development. They not only impact economic efficiency but also have direct implications for environmental quality and social welfare. The industrial structure and environmental policy management in Aba Prefecture, while promoting economic growth, also face complex environmental challenges. To achieve coordinated development between the economy and the environment, structural adjustments and policy optimization are required.The “Five-Year Plan” is an important strategic document formulated and implemented by the Chinese government for national economic and social development. By setting the plan, the government can outline the overall development direction, key tasks, and priorities, ensuring that the economy operates on a relatively stable trajectory. The “Five-Year Plan” typically includes goals aimed at promoting industrial structure adjustment and upgrading. For example, the plan may encourage the development of high-tech industries, green energy, or the service sector-measures that help enhance the overall competitiveness and sustainability of regional economies.

Industrial development is the fundamental driving force behind economic growth. The structure and scale of a region’s industries directly affect the speed, quality, and efficiency of economic development. Environmental pollution in a region is primarily caused by a low-level industrial structure [53], and the rapid development in Aba Prefecture during the post-disaster reconstruction has exacerbated the challenges of environmental governance.In 2011, the secondary industry in Aba Prefecture grew by 22.5%, contributing 65.4% to the economic growth and driving the economy up by 9.9 percentage points. However, from 2019 to 2020, the share of the tertiary industry surpassed that of the secondary industry, with the tertiary sector contributing 55.5% to economic growth. This shift reflects a transition in the economic structure, with a greater emphasis on services and higher-value-added sectors, signaling a gradual move towards more sustainable and diversified economic development.During the period from the 12th to the 13th Five-Year Plan, Aba Prefecture’s urbanization and economic structure continuously improved, and the per capita regional GDP saw steady growth. Notably, the tertiary sector, particularly tourism, experienced rapid development. In 2020, the number of tourists reached 21.28 million, generating ticket revenue of 521 million yuan.

The analysis of the Kuznets Curve between industrial wastewater discharge and per capita GDP indicates that with internal adjustments to the industrial structure, industrial wastewater emissions will gradually decrease. Looking ahead, it can be predicted that as the industrial structure continues to adjust, the ecological environment in Aba Prefecture will follow a positive development trajectory. However, it is essential to implement appropriate control measures for industrial emissions, such as dust and solid waste, to mitigate their environmental impact and ensure sustainable development.

From the experience of developed countries, it is found that investment in environmental protection should account for 1% to 1.5% of GDP to effectively control environmental pollution, and 3% is needed to achieve improvements in environmental quality. The central and provincial governments allocated special environmental protection funds to Aba Prefecture, which reached 20.1 million yuan in 2013. Of this, 6.48 million yuan (about 32%) was allocated for environmental pollution control, and 4.31 million yuan (about 21%) was used for environmental law enforcement, supervision, and monitoring capacity building. By 2021, the environmental protection special funds had increased to 15.205 million yuan.Building on this foundation, Aba Prefecture has focused on the goal of constructing “One Strong, One Land, and Two Zones” with ecological civilization as the core, intensifying public education and environmental monitoring efforts. Through the formulation and implementation of targeted policies and strict enforcement, Aba Prefecture has achieved certain successes in environmental protection.

## 7. Recommendations and limitations

Based on the current situation, Aba Prefecture needs to continue increasing investment in environmental protection in a targeted and sustainable manner [54] in order to ease the tension between economic development and environmental protection. While some reductions in pollution emissions have been achieved, the overall environmental governance measures are still insufficient, especially in areas such as domestic sewage and industrial solid waste. The lag in the construction of sewage treatment facilities has resulted in excessive domestic sewage discharge, failing to effectively alleviate the ecological burden.To address these shortcomings, Aba Prefecture needs to improve infrastructure, enhance the effectiveness of environmental policies, and accelerate the implementation of pollution control measures. This will be crucial in ensuring that economic growth and environmental sustainability can progress in tandem.Additionally, Aba Prefecture’s industries have not yet achieved full industrial upgrading and green transformation. There is a need to increase support for the development of new energy, clean technologies, and environmental industries. This can be done through fiscal subsidies, tax incentives, and other measures to encourage enterprises to invest in green production technologies, thus reducing industrial pollution.

Furthermore, although the Aba Prefecture government has implemented certain measures to control pollutant emissions, the enforcement of environmental protection policies remains weak. There is insufficient monitoring and penalties for pollution sources, which makes it difficult to fundamentally resolve pollution issues. To address this, the government needs to strengthen the implementation of environmental policies, raise environmental standards, and enhance oversight of polluting enterprises. Modern monitoring technologies should be employed to conduct real-time monitoring of pollutant emissions, ensuring that relevant enterprises comply with the law. Additionally, increasing public awareness of environmental protection, especially in rural areas and small- to medium-sized cities, through better promotion and education of environmental laws and regulations, will help facilitate the adoption of green lifestyles.

### 7.1 Policy implications recommendations

As a key ecological barrier and an important ecological function zone in China, Aba Prefecture faces a sensitive and fragile ecological environment[55]. To achieve long-term sustainable development, it is essential to drive the transformation of its industrial structure towards a higher level of ecological integration. A green technology development framework should be established, with the government playing a leading role and enterprises providing support, to introduce advanced ecological restoration and resource utilization technologies and foster green innovation[56]. This can be realized by setting up green technology research centers and funding related research, encouraging collaboration between universities, research institutions, and enterprises to tackle critical environmental technologies, particularly in water resource management, soil restoration, and solid waste treatment. Strengthening industrial investment is crucial, including the establishment of green technology incubators to support start-ups and research projects, and facilitating the commercialization of green technology innovations. Moreover, local region-specific industrial parks should be developed, with an emphasis on cooperation within the “Chengdu-Chongqing Twin City Economic Circle.” Simultaneously, the tourism industry should be revived through the “Tourism+” model, cultivating new business formats and brands. Cultural tourism must be prioritized as a strategic, leading, and pillar industry for Aba Prefecture, ensuring the alignment of economic, ecological, and social benefits.

Aba Prefecture needs to improve its environmental pollution control system and strengthen the cultivation and implementation of energy-saving and emission-reduction awareness[57]. The government should lead the implementation of the “Seven Protection Actions and Seven Governance Projects” and adhere to the system governance concept of “mountains, waters, forests, fields, lakes, grasslands, and deserts as a life community,” to enhance the stability of the ecological environment. A collaborative regulatory mechanism should be promoted to strictly control pollution emissions from key industries, enterprises, and particularly industrial “three wastes” (waste gas, waste water, and solid waste).In the short-term implementation plan, immediate efforts should be made to conduct pollution source investigations and establish environmental governance facilities, with a focus on advancing wastewater treatment and waste classification. Gradually, the goal should be to achieve full coverage of wastewater treatment facilities in administrative towns, while enhancing the management of urban wastewater treatment plants and expanding rural wastewater treatment infrastructure. Regular water quality monitoring and standardized operation and maintenance should be implemented to improve the service capacity of treatment facilities, ensuring strict adherence to the principles of “green development, safeguarding the baseline, following and protecting nature, focusing on priorities, implementing targeted measures, and local management with coordinated prevention and control.”In addition, based on the ecological conditions of different regions, an environmental access system for enterprises should be established to promote energy-saving and emission-reduction measures, the adoption of clean production technologies, and improved resource utilization. Comprehensive pollution investigation, monitoring, source tracing, and targeted governance should be conducted. Furthermore, multi-channel environmental protection and energy-saving knowledge dissemination activities should be carried out through schools, communities, and enterprises, particularly in rural areas, to enhance the environmental awareness of society. It is also important to encourage social organizations, volunteer groups, and enterprises to participate in environmental governance supervision, establish a reporting and reward mechanism, and create a positive atmosphere for public participation and joint governance in environmental protection [58].

Strengthen organizational leadership, integrate resources, and address prominent ecological and environmental issues in a systematic manner to promote the overall improvement of ecological and environmental quality [59]. The environmental challenges faced by Aba Prefecture are significant, but through precise policy design and effective implementation, the contradiction between economic development and environmental protection can be gradually resolved.In terms of fiscal resources, Aba Prefecture needs to increase government investment in environmental protection, particularly in supporting infrastructure development, technological research, and green projects. Multiple funding channels, such as local government funds, central government grants, and green finance, can be utilized to meet the financial needs of policy implementation.In terms of technology and human resources, Aba Prefecture should introduce advanced environmental protection technologies and equipment, especially in areas such as wastewater treatment, solid waste management, and clean energy utilization. At the same time, cooperation with research institutions, universities, and environmental protection enterprises should be strengthened to promote the local application of green technologies. Additionally, efforts should be made to train and recruit environmental professionals, enhancing the capacity for environmental monitoring, assessment, and management[60].

Through the implementation of the above suggestions, environmental quality can be improved, sustainable economic development can be achieved, and social welfare can be enhanced. A series of environmental protection policies will lead to significant improvements in air quality, water quality, soil, and other environmental factors in Aba Prefecture. Clean water sources and fresh air will greatly improve residents’ quality of life and reduce the negative health impacts of environmental pollution. The rapid development of green industries will bring new economic growth opportunities to Aba, driving employment, fostering technological innovation, and promoting industrial upgrades, which will enhance Aba’s competitiveness both nationally and globally.As environmental awareness increases and green lifestyles become more widespread, residents’ health levels will improve, and quality of life and social welfare will further rise. The implementation of environmental protection measures will reduce health issues and social costs caused by pollution, thereby promoting long-term social stability and harmony[61].

### 7.2 Limitations and future research recommendations

This study primarily focuses on Aba Prefecture, a region with unique economic development patterns, environmental policies, and geographical characteristics. These factors may limit the generalizability of the findings. Future research could address this limitation by conducting validation studies in similar regions or by incorporating Aba Prefecture as part of a case study to explore the prevalence of similar phenomena across broader regions. Additionally, while this study analyzed key influencing factors, it did not fully account for the impacts of potential underlying factors. Future studies could optimize the selection of indicators, such as including sulfur dioxide (SO₂) and carbon dioxide (CO₂) emissions, and integrate knowledge and methods from multiple disciplines such as economics, environmental science, and geography. This approach could provide a more accurate understanding of the complex relationship between economic development and environmental changes.

## Supporting information

S1 File Basic situation of environment and economic development from 2010 to 2021. (XLS)
